# Assessment of Physician Prescribing of Muscle Relaxants in the United States, 2005-2016

**DOI:** 10.1001/jamanetworkopen.2020.7664

**Published:** 2020-06-24

**Authors:** Samantha E. Soprano, Sean Hennessy, Warren B. Bilker, Charles E. Leonard

**Affiliations:** 1 Center for Pharmacoepidemiology Research and Training, Department of Biostatistics, Epidemiology, and Informatics, Perelman School of Medicine, University of Pennsylvania, Philadelphia; 2 Center for Clinical Epidemiology and Biostatistics, Department of Biostatistics, Epidemiology, and Informatics, Perelman School of Medicine, University of Pennsylvania, Philadelphia; 3 Center for Therapeutic Effectiveness Research, Department of Biostatistics, Epidemiology, and Informatics, Perelman School of Medicine, University of Pennsylvania, Philadelphia; 4 Department of Systems Pharmacology and Translational Therapeutics, Perelman School of Medicine, University of Pennsylvania, Philadelphia; 5 Neuropsychiatry Section, Department of Psychiatry, Perelman School of Medicine, University of Pennsylvania, Philadelphia

## Abstract

**Importance:**

Little is known to date about national trends in the prescribing of skeletal muscle relaxants (SMRs), the use of which is associated with important safety concerns, especially in older adults and in those who use concomitant opioids.

**Objective:**

To measure national trends in SMR prescribing over a 12-year period.

**Design, Setting, and Participants:**

This cross-sectional study used data from the National Ambulatory Medical Care Survey from January 2005 to December 2016. Data were analyzed from August 21, 2018, to July 18, 2019. The study included patients with ambulatory care visits who had encounters with non–federally funded, office-based physicians in the United States.

**Exposures:**

SMR use, categorized as newly prescribed or continued therapy at the office visit.

**Main Outcomes and Measures:**

Ambulatory care visits—overall and stratified by calendar year, geographic region, and patient age, sex, and race—in which an SMR was newly prescribed or continued were quantified. Among office visits in which an SMR was newly prescribed, diagnoses were assessed. Concomitant medications were quantified for all office visits, stratified by new or continued therapy. Survey visit weights were used to estimate nationally representative measures, and age-standardized rates were generated by geographic region using US Census data.

**Results:**

This study included a total of 314 970 308 office visits (mean [SD] age, 53.5 [15.2] years; 194 621 102 [61.8%] men and 120 349 206 [38.2%] women). In 2016, there were 30 730 262 (95% CI, 30 626 464-30 834 060) US ambulatory care visits in which an SMR was either newly prescribed or continued as ongoing therapy. Patients in these visits were most frequently female (58.2% [95% CI, 57.9%-58.6%]), white (53.7% [95% CI, 53.4%-54.0%]), and aged 45 to 64 years (48.5% [95% CI, 48.2%-48.9%]). During the study period, office visits with a prescribed SMR nearly doubled from 15.5 million (95% CI, 15.4-15.6 million) in 2005 to 30.7 million (95% CI, 30.6-30.8 million) in 2016. Although visits for new SMR prescriptions remained stable, office visits with continued SMR drug therapy tripled from 8.5 million (95% CI, 8.4-8.5 million) visits in 2005 to 24.7 million (95% CI, 24.6-24.8 million) visits in 2016. Older adults accounted for 22.2% (95% CI, 21.8%-22.6%) of visits with an SMR prescription. Concomitant use of an opioid was recorded in 67.2% (95% CI, 62.0%-72.5%) of all visits with a continuing SMR prescription.

**Conclusions and Relevance:**

This study found that SMR use increased rapidly between 2005 and 2016, which is a concern given the prominent adverse effects and limited long-term efficacy data associated with their use. These findings suggest that approaches are needed to limit the long-term use of SMRs, especially in older adults, similar to approaches to limit long-term use of opioids and benzodiazepines.

## Key Points

**Question:**

What trends characterize the outpatient prescribing of skeletal muscle relaxants in the United States?

**Findings:**

In this cross-sectional study of US physician visits, skeletal muscle relaxant prescribing doubled between 2005 and 2016. This increase was associated primarily with an increase in office visits with continuing use of skeletal muscle relaxants. New use of skeletal muscle relaxants was stable. Skeletal muscle relaxants were prescribed disproportionately to older adults, a high-risk population in whom these medications are potentially inappropriate, and were often prescribed concomitantly with opioids despite guidelines warning against this potentially dangerous combination.

**Meaning:**

This evidence of increasing continuing use of skeletal muscle relaxants, their disproportionately high use in older adults, and their concomitant use with opioids all represent trends with potentially adverse clinical and public health consequences.

## Teaser

This cross-sectional study uses data from the National Ambulatory Medical Care Survey from 2005-2016 to assess continued and new physician prescribing of muscle relaxants in the United States.

## Introduction

In response to the opioid epidemic, clinicians and patients are increasingly seeking alternatives to opioids for the management of musculoskeletal conditions. Centrally acting skeletal muscle relaxants (SMRs), such as baclofen, carisoprodol, chlorzoxazone, cyclobenzaprine, metaxalone, methocarbamol, orphenadrine, and tizanidine, are labeled for acute musculoskeletal conditions including spasms and lower back pain; they are used off-label for neuropathic pain, chronic noncancer pain, temporomandibular disorder pain, and numerous nonpain conditions.^1^ A 2003 systematic review concluded that SMRs are effective for acute low back pain (although their comparative effectiveness vs analgesics or nonsteroidal anti-inflammatory drugs for acute low back pain is unknown), the evidence for chronic low back pain is less convincing, and SMRs must be used with caution because of central nervous system adverse effects, such as drowsiness and dizziness.^2^ Because of the lack of evidence regarding the long-term efficacy and safety of SMRs and the unquantified risk of abuse, dependence, and overdose,^1,3^ recommendations generally limit the use of SMRs to a maximum duration of 2 to 3 weeks.^4^ Despite such recommendations, a 1988-1994 study found that 44.5% of people taking SMRs were continuously treated for longer than 1 year.^5^ Carisoprodol, chlorzoxazone, cyclobenzaprine, methocarbamol, metaxalone, and orphenadrine are all considered potentially inappropriate medications in older adults,^6,7^ in whom these agents are associated with sedation, cognitive impairment, and fracture.^8^ An additional concern regarding the inappropriate use of SMRs is the potential for drug-drug interactions, particularly with opioids.^9^

We sought to characterize national trends in SMR prescribing, both overall and in older adults, and to examine the concomitant prescribing of SMRs with opioids. Therefore, we examined nationally representative data from the National Ambulatory Medical Care Survey (NAMCS) for the 12-year period spanning from 2005 to 2016.

## Methods

We conducted a retrospective cross-sectional analysis of SMR prescribing using publicly available NAMCS data from January 2005 to December 2016. NAMCS is a US-based, annual survey of non–federally funded office-based physicians engaged in direct patient care.^10^ Survey data are collected from sampled health care professionals by trained proctors. Office visit records are weighted based on the most recently available census data to provide a nationally representative view of all ambulatory care visits in the United States. The survey captures information about the office visit, such as the reason for the encounter and diagnoses, medications, and demographic information about the patient, as well as information about the provider and their practice.

We identified all office visits in which an SMR was recorded as either newly prescribed or continued ongoing drug therapy, referred to herein as an SMR visit. To limit the data set to records of interest, we generated a list of Lexicon Plus (Cerner Multum Inc) drug identification codes for baclofen, carisoprodol, chlorzoxazone, cyclobenzaprine, metaxalone, methocarbamol, orphenadrine, and tizanidine. From these records, we extracted visit information, patient demographic characteristics, and record weights to generate national estimates. We examined the total number of visits per year; the race, ethnicity, and sex of the patient; and the region of the visit. Furthermore, we stratified counts by SMR agent and whether the SMR was newly prescribed or continued drug therapy.

We identified patients who were newly prescribed an SMR during the recorded visit by linking the new or continued status of reported medications (NCMed) variable to the SMR drug identification code. The NCMed variable indicates whether the medication was newly prescribed during the office visit or the patient was instructed to continue the medication as a part of their ongoing drug therapy. We examined the number of visits per year, the patient’s primary reason for the office visit, and all recorded diagnoses. All concomitant medications were examined for new SMR visits, and concomitant opioids were examined for continued SMR visits. A list of variables used, corresponding NAMCS variable names, and the population in which they were examined are presented in eTable 1 in the Supplement. For variables permitting multiple entries per visit, we included all entries without regard to ordering, eg, using all 5 diagnosis fields recorded in the 2016 survey.

We conducted analyses using the Statistical Package for Social Scientists, version 25 (IBM Corp) from August 21, 2018, to July 18, 2019. The University of Pennsylvania’s Office of Regulatory Affairs determined that this research did not require institutional review board oversight because the NAMCS is a publicly available data set. This article complies with the Strengthening the Reporting of Observational Studies in Epidemiology (STROBE) reporting guideline for cross-sectional studies.^11^

## Results

**Figure 1.**
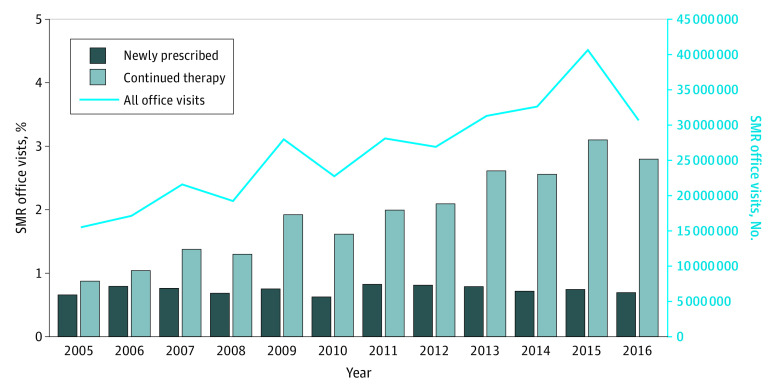
National SMR Utilization Stratified by New vs Continued Use, 2005-2016 SMR indicates skeletal muscle relaxant.

Patient demographic characteristics for SMR visits are shown in Table 1. The cross-sectional analysis included a total of 314 970 308 office visits (mean [SD] age, 53.5 [15.2] years; 194 621 102 [61.8%] men and 120 349 206 [38.2%] women). In 2016, there were 30 730 262 (95% CI, 30 626 464-30 834 060) US ambulatory care visits in which an SMR was either newly prescribed or continued as ongoing therapy. Patients prescribed an SMR in 2016 tended to be female (58.2% [95% CI, 57.9%-58.6%] women vs 41.8% [95% CI, 41.4%-42.1%] men). Racial demographic characteristics for SMR users in 2016 were as follows: 53.7% white (95% CI, 53.4%-54.0%), 10.2% African American (95% CI, 9.7%-10.6%), 1.2% Asian (95% CI, 1.0%-1.5%), and 2.2% Native American or Alaska Native (95% CI, 2.0%-2.5%). There were no data available for Native Hawaiians, and 1.5% (95% CI, 1.3%-1.8%) of patients had more than 1 race reported. As shown in eTable 2 in the Supplement and Figure 1, the number of US office visits in which an SMR was either newly prescribed or continued doubled from 15.5 million (95% CI, 15.4-15.6 million) visits in 2005 to 30.7 million (95% CI, 30.6-30.8 million) visits in 2016. During this 12-year period, the number of office visits resulting in new SMR prescriptions remained relatively stable at approximately 6 million (95% CI, 6.0-6.3 million) per year, whereas office visits for continued SMR drug therapy tripled from 8.5 million (95% CI, 8.4-8.5 million) to 24.7 million (95% CI, 24.6-24.8 million).

**Table 1.** Demographic Distribution^a^ of Patients in All SMR Office Visits, 2005-2016

Variable	Year											
	2005	2006	2007	2008	2009	2010	2011	2012	2013	2014	2015	2016
Sex												
Female	61.9 (61.5-62.3)	58.1 (57.7-58.3)	61.2 (60.9-61.4)	65.4 (65.0-65.6)	67.3 (67.0-68.5)	62.9 (62.7-63.1)	65.7 (65.5-65.9)	61.0 (60.9-61.1)	59.0 (58.9-59.1)	65.2 (65.0-65.2)	57.5 (54.2-60.7)	58.2 (57.9-58.6)
Male	38.1 (37.7-38.6)	41.9 (41.6-42.3)	38.9 (38.6-39.1)	34.6 (34.3-34.9)	32.7 (32.4-32.9)	37.1 (36.8-37.3)	34.3 (34.0-35.5)	38.9 (38.9-39.1)	41.0 (40.9-41.1)	34.8 (34.7-35.0)	42.5 (42.0-43.0)	41.8 (41.4-42.1)
Age, y												
<15	0.7 (0.4-1.1)	1.0 (0.6-1.4)	0.2 (0.00-0.4)	0.9 (0.7-1.1)	0.5 (0.3-0.8)	0.2 (0.1-0.2)	1.2 (09-1.3)	0.7 (0.1-1.5)	0.4 (0.3-0.5)	0.6 (0.6-0.7)	0.3 (0.2-0.4)	0.3 (0.1-0.5)
15-24	7.4 (7.0-7.8)	8.7 (8.4-9.1)	5.3 (5.0-5.6)	3.4 (3.1-3.7)	2.5 (2.3-2.7)	4.2 (3.9-4.5)	2.6 (2.3-2.8)	3.7 (3.6-3.8)	3.6 (3.5-3.7)	2.9 (2.2-3.1)	1.8 (1.4-2.2)	4.1 (3.7-4.4)
25-44	31.2 (30.8-31.7)	33.8 (33.4-34.0)	37.2 (36.8-37.4)	32.4 (32.1-32.7)	26.8 (26.6-27.0)	28.2 (27.9-29.3)	29.1 (28.9-29.3)	26.8 (26.7-26.9)	23.3 (23.1-23.3)	24.7 (24.6-24.8)	21.0 (20.6-21.3)	24.9 (24.6-25.2)
45-64	41.8 (40.9-42.0)	42.7 (42.2-43.0)	41.6 (41.3-41.8)	42.6 (42.3-42.9)	52.4 (52.2-52.7)	50.4 (50.2-50.7)	49.4 (49.1-49.6)	49.1 (49.0-49.2)	51.9 (51.8-52.0)	49.3 (48.6-49.5)	48.7 (48.3-49.0)	48.5 (48.2-48.9)
≥65	16.4 (15.9-16.8)	17.6 (16.2-17.9)	15.8 (15.4-16.0)	20.7 (20.4-21.0)	17.8 (17.5-18.3)	17.0 (16.8-17.3)	17.8 (17.6-17.9)	19.7 (19.6-19.7)	20.9 (20.7-21.0)	22.5 (22.4-22.6)	28.3 (27.8-28.7)	22.2 (21.8-22.6)
Race/ethnicity												
White	87.8 (87.4-88.2)	84.3 (83.9-84.6)	57.7 (57.5-57.9)	56.2 (55.9-56.5)	69.4 (69.1-69.7)	66.3 (66.0-66.8)	65.3 (65.0-65.4)	56.7 (56.6-56.8)	58.6 (58.5-58.7)	64.7 (64.5-64.8)	60.4 (60.1-60.8)	53.7 (53.3-54.0)
African American	10.3 (9.9-10.7)	12.7 (12.3-13.0)	10.3 (9.9-10.6)	10.1 (9.8-10.4)	11.2 (10.9-11.5)	13.7 (13.4-13.9)	8.8 (8.4-9.0)	7.9 (7.8-8.0)	10.8 (10.6-10.9)	7.7 (7.6-7.8)	11.5 (11.2-11.7)	10.2 (9.7-10.6)
Asian	1.4 (1.1-1.7)	1.3 (0.9-1.5)	1.0 (0.7-1.3)	1.1 (0.9-1.2)	0.7 (0.5-0.8)	0.3 (0.2-0.4)	1.7 (1.0-2.0)	1.1 (1.1-1.3)	1.8 (1.2-2.0)	2.2 (2.2-2.7)	0.4 (2.8-6.1)	1.2 (1.0-1.5)
Native Hawaiian	NR	0.9 (0.7-1.1)	NR	NR	NR	NR	NR	0.2 (0.0-0.3)	0.8 (0.0-0.1)	1.3 (1.0-1.4)	NR	NR
Native American or Alaska Native	NR	2.2 (0.5-1.3)	0.4 (0.1-0.5)	NR	0.1 (0.0-0.2)	0.2 (0.1-0.2)	0.4 (0.2-0.5)	0.3 (0.2-0.4)	0.2 (0.1-0.2)	0.7 (0.6-0.8)	0.2 (0.1-0.3)	2.2 (2.0-2.5)
>1 Race/ethnicity reported	NR	0.1 (0.0-0.1)	NR	0.7 (0.0-0.1)	0.3 (0.0-0.4)	NR	NR	0.3 (0.2-0.4)	0.2 (0.1-0.3)	0.3 (0.2-0.3)	NR	1.5 (1.3-1.8)

Abbreviations: NR, not reported; SMR, skeletal muscle relaxant.

^a^ Values are expressed as percentage (95% CI).

**Figure 2.**
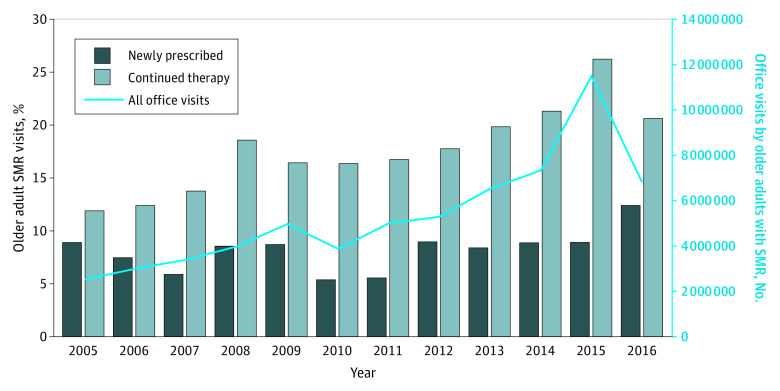
National SMR Utilization Rates Among Adults Aged 65 Years or Older, Stratified by New vs Continued Use, 2005-2016 SMR indicates skeletal muscle relaxant.

Adults older than 65 years accounted for 22.2% (95% CI, 21.9%-22.6%) of SMR visits in 2016, although this group accounted for just 14.5% of the US population.^12^ In 2016, the demographic characteristics of the other age groups were as follows: 0.3% (95% CI, 0.1%-0.5%) younger than 15 years, 4.1% (95% CI, 3.7%-4.4%) aged 15 to 24 years, 24.9% (95% CI, 24.6%-25.2%) aged 25 to 44 years, and 48.5% (95% CI, 48.2%-48.9%) aged 45 to 65 years. As shown in Figure 2, the proportion of visits that were SMR visits among patients 65 years and older increased 3-fold (from 1.3 SMR visits [95% CI, 1.0-1.7] per 100 office visits in 2005 to 4.3 SMR visits [95% CI, 4.1-4.6] per 100 office visits in 2016). The prescription of SMRs to older adults considered potentially inappropriate medications in this population (ie, carisoprodol, chlorzoxazone, cyclobenzaprine, metaxalone, methocarbamol, and orphenadrine) approximately doubled from 2.2 million (95% CI, 2.1-2.4 million) office visits in 2005 to 4.3 million (95% CI, 4.2-4.5 million) office visits in 2016.

The most common diagnoses between 2005 and 2015 reported for new SMR visits are shown in Table 2; the diagnosis coding system used for the NAMCS data shifted from the *International Classification of Diseases, Ninth Revision, Clinical Modification* to the *International Classification of Diseases, Tenth Revision, Clinical Modification* in 2016. The most common diagnoses during these visits were related to back pain and other musculoskeletal conditions; this pattern was maintained in 2016. As shown in Table 2, among new SMR visits, hydrocodone-acetaminophen was the most common concomitant therapy. Other analgesics, such as ibuprofen, naproxen, and tramadol, were also commonly used. As shown in eTable 3 in the Supplement, in 2016, 67.2% (95% CI, 62.0%-72.5%) of continuing SMR visits recorded concomitant therapy with an opioid, in contrast to 10.3% (95% CI, 9.8%-13.2%) of all ambulatory care visits nationally.

**Table 2.** Visit Diagnoses (2005-2015^a^) and Concomitant Medications (2005-2016) Among New SMR Visits

Variable	Office visits, No. (95% CI)
Diagnosis (*ICD-9-CM* code) (n = 78 671 742)^b^	
Other and unspecified disorders of back (724.9)	26 496 352 (25 635 583-27 357 121)
Sprains and strains of other and unspecified parts of back (847.9)	10 148 115 (9 559 679-10 736 551)
Spinal stenosis in cervical region (723.0)	8 205 661 (7 448 293-8 963 029)
Other disorders of soft tissues (729.99)	6 512 583 (5 996 830.56-7 028 335)
Essential hypertension (401.1)	6 049 615 (5 708 365-6 390 866)
Intervertebral disc disorders (722.90)	6 030 779 (5 425 636-6 635 922)
Disorders of muscle, ligament, and fascia (728.79)	4 585 666 (4 254 719-4 916 613)
Anxiety state (300.0)	3 167 519 (2 303 704-4 031 334)
Other and unspecified disorders of joint (719.98)	2 989 405 (2 688 389-3 290 421)
Disorders of lipoid metabolism (272.9)	2 694 600 (2 362 481-3 026 719)
Concomitant medication (n = 84 850 041)^c^	
Hydrocodone-acetaminophen	14 096 447 (13 380 518-14 812 376)
Ibuprofen	12 531 204 (12 004 039-13 058 369)
Naproxen	9 820 338 (9 321 411-10 319 265)
Tramadol	5 011 229 (4 680 556-5 341 902)
Lisinopril	4 208 202 (1 105 116-7 311 288)
Meloxicam	4 069 328 (3 556 643-4 582 013)
Aspirin	3 969 403 (3 649 823-4 288 983)
Omeprazole	3 927 795 (3 455 672-4 399 919)
Albuterol	3 781 380 (3 376 754-4 186 006)
Diclofenac	3 752 475 (3 545 994 -3 958 956)

Abbreviations: *ICD-9-CM*, *International Classification of Diseases, Ninth Revision, Clinical Modification*; SMR, skeletal muscle relaxant.

^a^ 2016 excluded because of transition to *International Classification of Diseases, Tenth Revision, Clinical Modification*.

^b^ Among all new SMR office visits from 2005 to 2015.

^c^ Among all new SMR office visits from 2005 to 2016.

Table 3 shows the number of office visits and age-standardized rates of SMR visits for 2005 and 2016 stratified by geographic region and by new vs continued SMR. In the Northeast, age-standardized new SMR visit rates changed by −33.4% (95% CI, −31.7% to −36.4%), whereas continued SMR visit rates increased by 325.1% (95% CI, 320.2% to 342.4%). We observed a similar pattern in the South, with a −15.8% (95% CI, −15.2% to −17.0%) change in new SMR visits accompanied by a 79.1% (95% CI, 68.8% to 82.2%) increase in continued SMR visits. In the Midwest, new and continued SMR visit rates increased by 26.9% (95% CI, 22.1% to 28.9%) and 297.6% (95% CI, 273.8% to 307.5%), respectively. We observed similar but less marked increases in new and continued SMR visit rates in the West, with increases of 5.4% (95% CI, 3.8% to 5.6%) and 91.6% (95% CI, 87.4% to 93.2%), respectively.

**Table 3.** 12-Year Change in SMR Utilization by Geographic Region, 2005-2016

Age group, y^a^	No. of office visits			Age-standardized rate per million population^b^		% Difference (95% CI)
	2005	2016	2005		2016	
**New SMR visits**						
Northeast	806 072	604 326	14 884		9917	
<18	59 485	NR	1142		NR	−33.4 (−31.7 to −36.4)
18-24	142 000	NR	2866		NR	
25-44	171 266	112 423	3156		2288	
45-64	410 995	356 956	7326		5690	
≥65	22 326	134 952	393		1938	
Midwest	1 308 190	1 755 480	20 073		25 466	
<18	56 558	NR	863		NR	26.9 (22.1 to 28.9)
18-24	122 191	111 327	1928		1667	
25-44	310 611	625 067	4845		10 681	
45-64	729 940	413 552	11 048		5642	
≥65	88 890	605 534	1390		7476	
South	3 100 314	2 433 079	22 997		19 362	
<18	248 784	68 892	2314		610	–15.8 (−15.2 to −17.0)
18-24	448 217	315 016	4391		2688	
25-44	984 557	201 379	9255		1872	
45-64	949 278	1 159 135	9181		9270	
≥65	469 478	688 656	4562		4922	
West	1 160 469	1 385 413	17 773		18 728	
<18	22 919	197 263.0	321		2752	5.4 (3.8 to 5.6)
18-24	71 311	153 745	1078		2042	
25-44	373 064	396 164	5300		5603	
45-64	537 379	612 761	8487		8020	
≥65	155 796	25 480	2587		311	
Overall US	6 375 045	6 178 298	22 017		18 855	–14.4 (−12.3 to −15.1)
**Continued SMR visits**						
Northeast	1 324 873	5 763 559	23 768		101 030	
<18	NR	NR	NR		NR	325.1 (320.2 to 342.4)
18-24	4466	242 937	90		44 441	
25-44	339 226	2 312 550	6252		168 064	
45-64	682 694	2 191 924	12 169		141 183	
≥65	298 487	1 016 148	5258		117 659	
Midwest	1 662 278	7 086 853	25 627		101 891	
<18	13 411	92 265	205		1461	297.6 (273.8 to 307.5)
18-24	5777	50 600	91		758	
25-44	787 613	2 106 184	12 286		35 989	
45-64	659 845	3 051 307	9987		41 626	
≥65	195 632	1 786 498	3058		22 057	
South	3 641 577	7 804 048	34 745		62 232	
<18	NR	NR	NR		NR	79.1 (68.8 to 82.2)
18-24	432	NR	4		NR	
25-44	1 133 092	11 341 547	10 651		10 544	
45-64	1 770 148	4 724 147	17 120		37 782	
≥65	717 800	1 945 345	6974		13 905	
West	1 822 159	4 086 159	28 189		54 004	
<18	46 457	40 390	650		563	91.6 (87.4 to 93.2)
18-24	NR	64 101	NR		852	
25-44	513 580	951 051	7296		13 451	
45-64	879 499	2 424 176	13 891		31 756	
≥65	382 623	606 442	6352		7411	
Overall US	8 450 887	24 740 620	29 144		75 430	158.8 (143.2 to 164.0)

Abbreviations: NR, not reported; SMR, skeletal muscle relaxant.

^a^ Regions defined by the US Census Bureau.

^b^ 2005 US population used as the reference group for age standardization.

## Discussion

This analysis of nationally representative office visit data found that SMR use doubled from 2005 to 2016 and that there was a disproportionately high use of these drugs in older adults, a population in which SMR use is potentially inappropriate. These increasing rates did not appear to have an association with the decline in opioid prescribing that began in 2012 in both the general ambulatory care population and in the older adult population.^13^ Furthermore, among visits with a continuing SMR, 67.2% of patients were concomitantly treated with an opioid—a combination that has the potential to cause serious drug-drug interactions, such as potentiated central nervous system depression and an increased risk of opioid overdose.^9^ As expected, among new users of SMRs, we found that the most common diagnoses were related to back pain and other musculoskeletal conditions. Interestingly, although the frequency of new initiation of SMR therapy remained stable, the number of office visits in which SMR therapy was continued tripled, indicating a potential shift in duration of use of these drugs. This trend is a concern given the limited evidence of long-term efficacy and the risk of serious central nervous system adverse effects and drug-drug interactions.

Although prior papers have examined the use of SMRs in veterans^4^ and older adults,^14,15^ the present study is, to our knowledge, the first study since 2004^5,16^ to examine SMR use in the general population. The strengths of this study stem from the design of the NAMCS that permits us to make projections to US physician office visits.

### Limitations

This study has some limitations. The NAMCS does not capture patients leaving the hospital with an SMR prescription or allow researchers to follow patients over time or assess clinical outcomes. In addition, these data are limited to the United States, and we are unaware of recent analogous data from other countries.

## Conclusions

Given their prominent adverse effects and the limited evidence for their long-term efficacy, growth in the continued use of SMRs, particularly in older adults and concomitantly with opioids, is concerning. Given the findings of this cross-sectional study, efforts to limit the long-term use of SMRs may be needed, especially for older adults, similar to efforts used to limit the long-term use of opioids^17^ and benzodiazepines.^18^

## Notes

Supplement. **eTable 1.** NAMCS Variable and Population of Analysis **eTable 2.** Number of US Office Visits with a Skeletal Muscle Relaxant Prescription, 2005-2016 **eTable 3. **Office Visits With Newly Prescribed or Continued Opioid Therapy, 2016
