# Supplementary material for: Assessment of Physician Prescribing of Muscle Relaxants in the United States, 2005-2016
Source: JAMA Netw Open. 2020 Jun 24;3(6):e207664. doi: 10.1001/jamanetworkopen.2020.7664 (PMC7315288; doi:10.1001/jamanetworkopen.2020.7664)
Supplement: Supplement. — eTable 1. NAMCS Variable and Population of Analysis eTable 2. Number of US Office Visits with a Skeletal Muscle Relaxant Prescription, 2005-2016 eTable 3. Office Visits With Newly Prescribed or Continued Opioid Therapy, 2016 [file jamanetwopen-3-e207664-s001.pdf]

Supplementary Online Content

Soprano SE, Hennessy S, Bilker WB, Leonard CE. Assessment of physician prescribing of muscle relaxants in the United States, 2005-2016. *JAMA Netw Open*. 2020;3(6):e207664. 10.1001/jamanetworkopen.2020.7664

eTable 1. NAMCS Variable and Population of Analysis

eTable 2. Number of US Office Visits with a Skeletal Muscle Relaxant Prescription, 2005-2016

eTable 3. SMR Office Visits With Newly Prescribed or Continued Opioid Therapy, 2016

This supplementary material has been provided by the authors to give readers additional information about their work.

© 2020 Soprano SE et al. *JAMA Network Open.*

eTable 1. NAMCS Variable and Population of Analysis

Variable	Type	NAMCS variable name	Population
Patient Race	Demographics	RACER	All Patients
Patient Age	Demographics	AGER	All Patients
Patient Ethnicity	Demographics	ETHUN	All Patients
Patient Sex	Demographics	SEX	All Patients
Region of Visit	Demographics	REGIONOFF	All Patients
Major Reason for Visit	Visit Information	MAJOR	Patients newly prescribed SMRs
Diagnosis	Visit Information	2016: DIAG1-DIAG5 (ICD-10)	Patients newly prescribed SMRs
		2005-2015: DIAG13D-DIAG53D[a] (ICD-9)	
Drug ID	Prescribing Information	DRUGID1-DRUGID30[b]	Patients newly prescribed SMRs

Abbreviation: ICD, International Classification of Diseases; NAMCS, National Ambulatory Medical Care Survey.

[a]2005-2013 ended at DIAG33D (ICD-9); 2014-2015 ended at DIAG53D (ICD-9)

[b]2005-2011 ended at DRUGID8; 2012-2013 ended at DRUGID10; 2014-2016 ended at DRUGID30

© 2020 Soprano SE et al. *JAMA Network Open.*

eTable 2. Number of US Office Visits with a Skeletal Muscle Relaxant Prescription, 2005-2016

Year	No. of SMR visits (95% CI)	Office visits with an SMR, % (95% CI)
2005	15 549 220 (15 482 187-15 616 253)	1.6 (1.6-1.6)
2006	17 171 863 (17 115 009-17 228 716)	1.9 (1.9-1.9)
2007	21 619 272 (21 562 127-21 676 417)	2.2 (2.2-2.2)
2008	19 271 770 (19 212 842-19 330 698)	2.0 (2.0-2.1)
2009	28 018 540 (27 949 194-28 087 886)	2.7 (2.7-2.7)
2010	22 795 511 (22 738 912-22 852 109)	2.3 (2.2-2.3)
2011	28 152 491 (28 087 765-28 217 216)	2.9 (2.8-2.9)
2012	26 961 188 (26 940 140-26 982 235)	2.9 (2.9-2.9)
2013	31 346 196 (31 308 619-31 383 771)	3.4 (3.4-3.4)
2014	32 664 042 (32 625 467-32 702 616)	3.7 (3.6-3.7)
2015	40 689 953 (40 527 486-40 852 419)	4.1 (4.0-4.1)
2016	30 730 262 (30 626 464-30 834 060)	3.5 (3.4-3.5)

Abbreviation: CI, confidence interval; SMR, skeletal muscle relaxant; US, United States.

© 2020 Soprano SE et al. *JAMA Network Open.*

eTable 3. SMR Office Visits With Newly Prescribed or Continued Opioid Therapy, 2016

Active ingredient	Drug ID	SMR visit population	Continued SMR visit population	General ambulatory care population
Codeine	d03357	107 515	-	991 236
Codeine phosphate	d03393	227 261	227 261	2 515 977
Codeine phosphate	d03398	-	-	31 445
Codeine phosphate	d03423	735 432	590 946	5 387 802
Codeine phosphate	d03425	266 211	266 211	500 251
Codeine sulfate	d00012	-	-	368 769
Fentanyl citrate	d00233	798 528	798 528	3 276 591
Hydrocodone bitartrate	d03075	729 589	689 452	5 138 126
Hydrocodone bitartrate	d03340	-	-	103 143
Hydrocodone bitartrate	d03353	-	-	47 556
Hydrocodone bitartrate	d03356	-	-	182 936
Hydrocodone bitartrate	d04225	-	-	95 484
Hydrocodone-acetaminophen	d03428	5 974 022	5 245 540	27 531 878
Hydromorphone hydrochloride	d00255	91 011	91 011	729 092
Meperidine hydrochloride	d00017	45 729	45 730	380 998
Methadone hydrochloride	d00050	-	-	971 308
Morphine sulfate	d00308	880 448	852 493	3 573 695
Opium tincture	d00824	118 036	118 036	219 235
Oxycodone hydrochloride	d00329	2 102 502	2 028 847	10 756 882
Oxycodone hydrochloride	d03431	1 249 561	1 195 804	8 353 788
Oxycodone hydrochloride	d03432	53 758	-	127 960
Oxymorphone hydrochloride	d00833	155 914	155 914	283 922
Pentazocine lactate	d00334	-	-	235,318

© 2020 Soprano SE et al. *JAMA Network Open.*

Tapentadol (as hydrochloride)	d07453	47 071	47 071	68 659
Tramadol hydrochloride	d03826	4 789 305	4 278 706	18 791 920
Tramadol hydrochloride	d04766	-	-	192 500
Total		18 371 891	16 631 547	90 856 468
Population, % (95% CI)		59.7 (56.8-61.3)	67.2 (62.0-72.4)	10.3 (9.8-13.2)

Abbreviations: CI, confidence interval; ID, identification; SMR, skeletal muscle relaxant.

© 2020 Soprano SE et al. *JAMA Network Open.*
